# Factors associated with clinically diagnosed long QT syndrome in Korean adults: a nationwide case-control study

**DOI:** 10.3389/fphar.2026.1904560

**Published:** 2026-08-20

**Authors:** Dong-Young Park, Sangmin Park, Jin-Won Kwon, Yun-Kyoung Song

**Affiliations:** 1 College of Pharmacy, The Catholic University of Korea, Bucheon, Republic of Korea; 2 BK21 FOUR Community-Based Intelligent Novel Drug Discovery Education Unit, College of Pharmacy and Research Institute of Pharmaceutical Sciences, Kyungpook National University, Daegu, Republic of Korea

**Keywords:** Asian population, case-control studies, drug safety, Korean adults, long QT syndrome, pharmacoepidemiology, QT-prolonging drugs

## Abstract

**Introduction:**

Long QT syndrome (LQTS) is a major substrate for malignant ventricular arrhythmias, yet population-level evidence on its clinical, pharmacological and metabolic correlates in Asian adults remains limited.

**Methods:**

Using Korean National Health Insurance Service claims linked to national health screening data (2012–2022), we conducted a 1:1 age- and sex-matched case-control study to characterize factors associated with LQTS in a nationwide adult population. Cases were identified by LQTS-related diagnostic codes with concurrent electrocardiographic procedure claims, and controls were individuals without any arrhythmia diagnosis. Adjusted odds ratios (aORs) with 95% confidence intervals were estimated by multivariable conditional logistic regression for comorbidities, QT-prolonging drug exposure, and metabolic and laboratory factors, with prespecified subgroup analyses by sex and age.

**Results:**

The study included 335,693 cases and 335,693 matched controls (mean age 54.6 years; 48.6% male). Cardiovascular comorbidities, including chronic heart failure and peripheral arterial disease, were among the factors most strongly associated with LQTS. The strength of association increased progressively with the number of concurrently used QT-prolonging drugs, with aORs of 6.70, 11.63 and 15.08 for one, two, and three or more drugs, respectively. Hyperkalaemia, calcium imbalance, central obesity, elevated blood pressure, dyslipidaemia and impaired renal function were also independently associated with LQTS. Associations with hyperkalaemia and thyroid dysfunction were numerically stronger in women, and the drug-related gradient was steepest in older adults.

**Discussion:**

These nationwide findings characterize the comorbidity, electrolyte and cumulative drug-exposure factors associated with clinically diagnosed LQTS in Korean adults, and may complement Western-derived tools such as CredibleMeds to inform population-specific pharmacovigilance of QT-prolonging drugs.

## Introduction

The QT interval on electrocardiography reflects the duration of ventricular depolarization and repolarization and serves as a marker of cardiac electrical stability. Prolongation of the heart rate-corrected QT interval (QTc) indicates delayed ventricular repolarization and is a well-established risk factor for Torsades de pointes, which can progress to ventricular fibrillation and sudden cardiac death (Schwartz, Crotti and Insolia, 2012). Long QT syndrome (LQTS) affects approximately one in 2,500 individuals and, when untreated, carries a clinically important risk of sudden cardiac death (Viskin, 2020).

While pathogenic genetic variants drive congenital LQTS, acquired factors including comorbidities, concomitant use of QT-prolonging drugs, and lifestyle factors such as obesity and smoking also contribute substantially to QTc prolongation (Roden, 2004; Straus et al., 2006). Clinical tools such as CredibleMeds have been widely used to identify drug-induced LQTS (Then et al., 2022), but they cannot fully account for the interplay of comorbidities and demographics, nor do they provide a quantitative assessment of the cumulative risk associated with concurrent use of multiple QT-prolonging drugs (Agnihotri et al., 2024).

Ethnic differences in QT prolongation and its associated mortality have been reported. Asian populations differ from Western counterparts in genetic background, lifestyle, and cardiovascular disease risk profiles, which may influence QT intervals and related outcomes (Seyerle et al., 2014). Susceptibility to drug-induced QT interval prolongation also varies by ethnicity (Niemeijer et al., 2015). However, most prior investigations of QT prolongation risk factors have been conducted in Western cohorts (Whitsel et al., 2005; Eroglu et al., 2022), and existing studies in Asian populations have generally been limited by single-centre designs, drug-specific comparative analyses, or highly selected patient populations (Choi et al., 2018).

This gap highlights the need for population-based studies of LQTS risk factors in Asian populations to inform prevention and management. We therefore conducted a nationwide, 1:1 age- and sex-matched case-control study using linked Korean national claims and health-screening data to characterize a broad spectrum of factors associated with clinically diagnosed LQTS, spanning comorbidities, cumulative QT-prolonging drug exposure, and metabolic, laboratory and lifestyle parameters, with prespecified subgroup analyses by sex and age. By providing population-specific estimates from a large Asian adult cohort, this study may complement decision-support tools such as CredibleMeds, which were largely derived from Western populations.

## Materials and methods

### Data source

This retrospective, population-based case-control study used health insurance and general health check-up data from 2012 to 2022, provided by the National Health Insurance Service (NHIS) of Korea. The NHIS claims database includes demographic characteristics, diagnoses based on the International Classification of Diseases, 10th Revision (ICD-10), prescription medications (drug components, prescription dates, and dosages), medical procedures, and hospitalization records (Cheol Seong et al., 2017). The linked National Health Screening Database provides anthropometric measurements (body mass index, waist circumference), blood pressure, laboratory test results (fasting glucose, total cholesterol, high-density lipoprotein and low-density lipoprotein cholesterol, triglycerides, and liver function parameters), and lifestyle factors obtained via standardized questionnaires, including smoking status. Korea’s National Health Screening Program conducts biennial examinations for all health insurance beneficiaries and dependents aged 20 years and older (Seong et al., 2017). The study was approved, with a waiver of informed consent, by the Institutional Review Board of Daegu Catholic University (IRB No. CUIRB-2023-E003; see Ethics approval).

### Study population

Potential cases and controls were identified from the NHIS database based on the presence or absence of arrhythmia diagnoses (ICD-10 codes I45, I47, and I49) between 2012 and 2022. Cases were defined as individuals with LQTS, identified by at least one relevant diagnostic code (I45.8 prolongation of QT interval; I49.8 long QT syndrome; I47.2 torsades de pointes; or I49.0 ventricular fibrillation and flutter), together with concurrent or temporally related claims for electrocardiographic procedure codes (E6544, bedside ECG monitoring per day; E6545, 24-h Holter ECG monitoring; or E6556, Holter ECG monitoring for more than 48 h and up to 7 days), indicating that a multiple electrocardiographic assessment was performed (Ye, Larrat and Caffrey, 2018). These qualifying codes correspond to continuous or ambulatory (Holter) monitoring rather than a single point-in-time recording, capturing repolarisation over an extended period. Controls were drawn from individuals without any arrhythmia diagnosis in the NHIS database and were matched 1:1 to cases on sex and exact age. Each control was assigned the index date of its matched case, defined as the date of LQTS diagnosis. This design, which contrasts clinically diagnosed cases with an arrhythmia-free reference population, was intended to identify and rank factors associated with LQTS rather than to estimate absolute risk. Individuals with incomplete or inconsistent data were excluded.

### Exposure of interest

Potential factors associated with LQTS were identified based on prior evidence and clinical relevance and grouped into three major categories: comorbidities, drugs, and clinical parameters comprising laboratory values and lifestyle factors (Vandael et al., 2017). The comorbidities considered were hypertension, myocardial infarction, chronic heart failure, diabetes mellitus, chronic kidney disease, thyroid dysfunction, cancer, cerebrovascular disease, peripheral arterial disease, and electrolyte imbalances of magnesium, potassium, and calcium, with the corresponding ICD-10 definitions listed in Supplementary Table S1. These comorbidities were ascertained identically in cases and controls from diagnostic records within the 6 months preceding the index date (Quan et al., 2005), following the covariate assessment timeline shown in Supplementary Figure S1. Exposure to the QT-prolonging drugs detailed in Supplementary Table S2 during the 1 month preceding the index date was evaluated for both cases and their matched controls. The number of concurrently administered QT-prolonging drugs was analyzed as an exposure to capture potential cumulative or interactive effects (Roden, 2008). Clinical laboratory and lifestyle factors were defined using the most recent health check-up data available prior to the index date and included body mass index, waist circumference, blood pressure, fasting glucose, hemoglobin, lipid profile, hepatic and renal function indicators, and smoking status.

### Statistical analysis

Categorical variables were summarized as frequencies and percentages, and continuous data as means with standard deviations, with standardized mean differences used for comparison (Austin, 2009). Associations between candidate factors and LQTS were estimated using multivariable conditional logistic regression, accounting for the 1:1 matched design (Breslow and Day, 1980). For each factor of interest, all remaining covariates were simultaneously included as adjustment variables, and associations were expressed as adjusted odds ratios (aORs) with 95% confidence intervals. Missing values in health screening-based clinical parameters were addressed using multiple imputation by chained equations under a missing-at-random assumption, generating [m] imputed datasets that were combined using Rubin’s rules (White, Royston and Wood, 2011). Subgroup analyses stratified by sex and by age group, namely <40, 40–64, and ≥65 years, were performed using multivariable logistic regression; sex-stratified models were additionally adjusted for age and age-stratified models for sex, as the original 1:1 matching structure was not preserved within each stratum.

A Sankey diagram was constructed to visualize patterns of co-occurrence among disease history, medication use, and case–control status. Patient-level data were aggregated by diseases and medications, and node-link structures were organized by case and control groups. Node size was proportional to the number of patients, while link thickness reflected the frequency of the corresponding disease or medication pathway. Red-scale flows represented case-related pathways and blue-scale flows represented control-related pathways. All analyses were performed using SAS Version 9.3 (SAS Institute, Cary, NC, United States) and Python 3.11.

## Results

### Baseline characteristics of the study population

**FIGURE 1:**
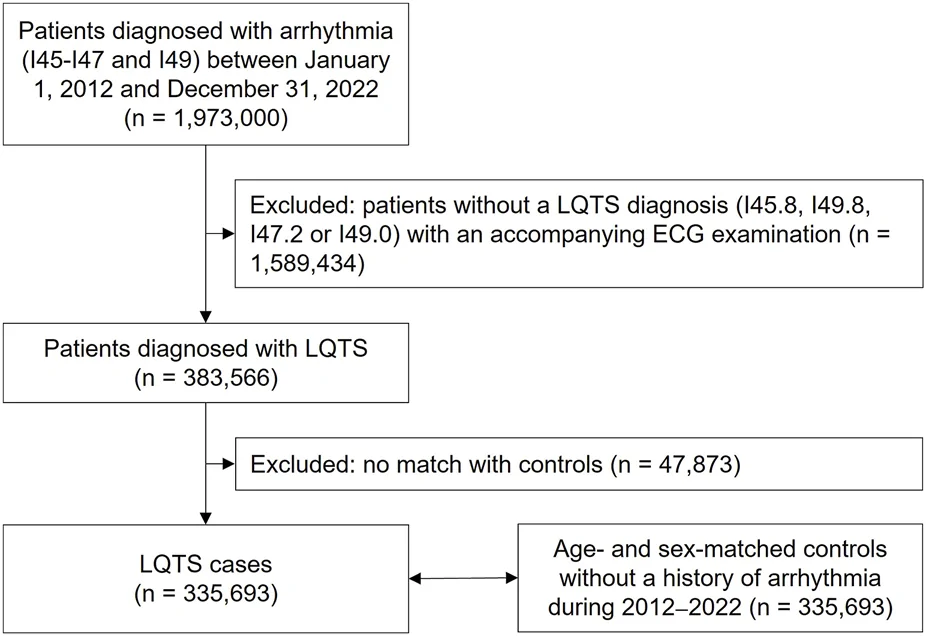
Study population selection flowchart. Flow of participant selection from the National Health Insurance Service database. From 1,973,000 individuals with arrhythmia diagnoses (ICD-10 codes I45, I47, and I49) recorded between 2012 and 2022, those without long QT syndrome (LQTS)-specific diagnostic codes and those without suitable matches were excluded, yielding 335,693 cases. An equal number of controls without any arrhythmia diagnosis were matched 1:1 on sex and age, with each control assigned the index date of its matched case. ICD-10, International Classification of Diseases, 10th Revision; LQTS, long QT syndrome.

From an initial 1,973,000 individuals with arrhythmia diagnoses between 2012 and 2022, those without LQTS-specific diagnostic codes and those without suitable matches were excluded, yielding 335,693 cases; an equal number of controls without any arrhythmia diagnosis were then matched 1:1 to the cases. The final study population comprised 335,693 cases and 335,693 controls matched on sex and age, with each control assigned the index date of its matched case; the mean age was 54.6 years and 48.6% were male in both groups, as shown in Figure 1 and Supplementary Table S3.

### Comorbidities and QT-prolonging drug profiles

**FIGURE 2:**
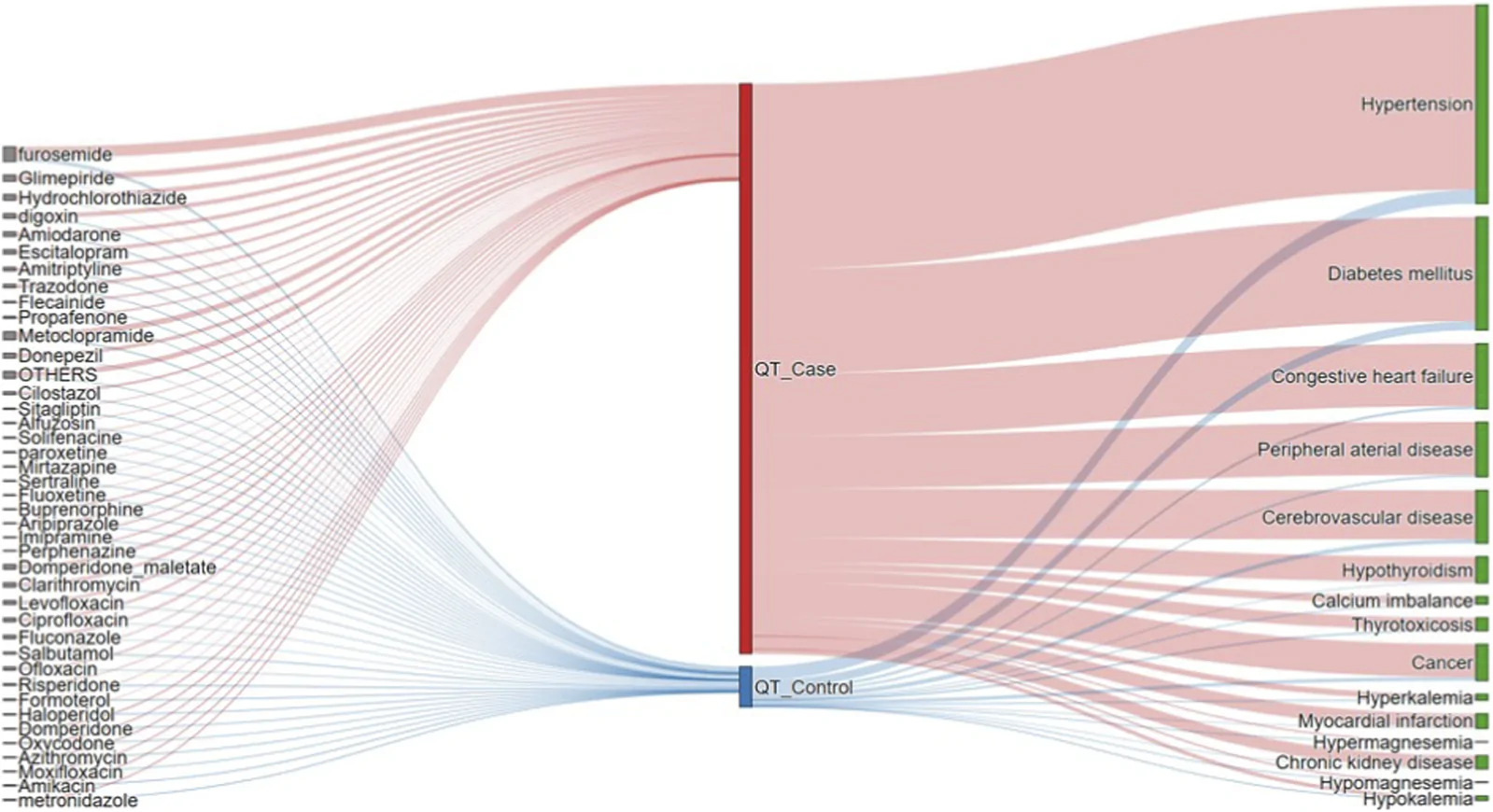
Sankey diagram of disease-medication flow patterns in LQTS cases and controls. Nodes represent comorbidities, QT-prolonging medications, and case or control status; node size is proportional to the number of patients, and link thickness reflects the frequency of the corresponding disease or medication pathway. Red-scale flows denote case-related pathways and blue-scale flows denote control-related pathways. LQTS, long QT syndrome.

The Sankey diagram in Figure 2 illustrates the frequency of individual QT-prolonging drugs and comorbidities in cases and controls. Within cases, hypertension, chronic heart failure, diabetes mellitus, peripheral arterial disease, and cerebrovascular disease were the predominant comorbidities, and patients with these conditions exhibited higher rates of QT-prolonging drug use. Medication patterns in cases were characterized by prominent use of diuretics such as furosemide and hydrochlorothiazide, antiarrhythmics such as amiodarone and propafenone, antipsychotics such as haloperidol and risperidone, antidepressants such as escitalopram and fluoxetine, antibiotics such as levofloxacin and ciprofloxacin, and the antifungal fluconazole. By contrast, controls showed markedly lower frequencies of both comorbidities and QT-prolonging drug use. Cases clustered around specific comorbidities and showed overlapping patterns of QT-prolonging drug exposure, indicating frequent co-occurrence of these comorbidities and medications.

### Factors associated with LQTS

As shown in Table 1, cardiovascular, endocrine, oncologic, and electrolyte-related disorders were all significantly associated with LQTS. The strongest associations were observed for chronic heart failure (aOR 22.31, 95% confidence interval [CI] 21.28–23.38) and peripheral arterial disease (aOR 21.65, 95% CI 20.56–22.80). Within endocrine disorders, hypothyroidism showed a particularly strong association (aOR 17.34, 95% CI 16.16–18.62). Electrolyte abnormalities were also strongly associated with LQTS, especially calcium imbalance (aOR 18.17, 95% CI 15.89–20.77), hypermagnesaemia (aOR 15.99, 95% CI 9.30–27.51), and hyperkalaemia (aOR 15.20, 95% CI 13.20–17.51).

**TABLE 1:** Associations between comorbidities and risk of long QT syndrome.

Comorbidity	Cases (n = 335,693), n (%)	Controls (n = 335,693), n (%)	Crude odds ratio (95% CI)	Adjusted odds ratio (95% CI)
Hypertension	154,771 (46.1)	11,509 (3.4)	24.10 (23.62–24.58)	19.59 (19.16–20.03)
Myocardial infarction	11,463 (3.4)	854 (0.3)	13.86 (12.93–14.86)	10.53 (9.71–11.41)
Chronic heart failure	52,272 (15.6)	2,215 (0.7)	27.77 (26.60–28.98)	22.31 (21.28–23.38)
Cerebrovascular disease	40,953 (12.2)	3,564 (1.1)	12.95 (12.51–13.40)	9.66 (9.28–10.06)
Peripheral arterial disease	44,004 (13.1)	1,818 (0.5)	27.71 (26.43–29.04)	21.65 (20.56–22.80)
Diabetes mellitus	87,121 (26.0)	7,421 (2.2)	15.50 (15.13–15.88)	12.31 (11.97–12.67)
Hypothyroidism	21,076 (6.3)	993 (0.3)	22.58 (21.18–24.07)	17.34 (16.16–18.62)
Thyrotoxicosis	10,356 (3.1)	636 (0.2)	16.77 (15.48–18.17)	12.88 (11.76–14.11)
Cancer	27,801 (8.3)	3,006 (0.9)	9.99 (9.62–10.38)	7.25 (6.93–7.59)
Chronic kidney disease	10,440 (3.1)	797 (0.2)	13.49 (12.55–14.50)	10.32 (9.49–11.22)
Hypermagnesaemia	351 (0.1)	17 (<0.1)	20.67 (12.70–33.63)	15.99 (9.30–27.51)
Hypomagnesaemia	496 (0.2)	42 (<0.1)	11.83 (8.63–16.20)	8.64 (5.96–12.53)
Hyperkalaemia	5,015 (1.5)	253 (0.1)	20.11 (17.72–22.82)	15.20 (13.20–17.51)
Hypokalaemia	3,470 (1.0)	357 (0.1)	9.81 (8.80–10.94)	6.69 (5.86–7.64)
Calcium imbalance	6,274 (1.9)	273 (0.1)	23.40 (20.73–26.42)	18.17 (15.89–20.77)

Abbreviations: OR, odds ratio; aOR, adjusted odds ratio; CI, confidence interval.

As shown in Table 2, the number of QT-prolonging drugs was strongly associated with LQTS. Patients exposed to at least one QT-prolonging drug accounted for 15.9% of cases compared with 3.8% of controls. The strength of association increased with the number of concurrently administered drugs, with aORs of 6.70 (95% CI 6.25–7.17) for one drug, 11.63 (95% CI 10.16–13.30) for two drugs, and 15.08 (95% CI 11.79–19.30) for three or more. Most QT-prolonging drug classes were associated with significantly elevated odds of LQTS, as detailed in Supplementary Table S4; antiarrhythmic agents showed the highest association (aOR 9.29, 95% CI 8.56–10.10), followed by antidepressants (aOR 8.47, 95% CI 7.96–9.01) and urologic agents (aOR 7.80, 95% CI 6.80–8.94). Among the health check-up parameters, central obesity, elevated blood pressure on screening, dyslipidaemia, and renal dysfunction showed strong associations with LQTS, with aORs ranging from 4.56 to 21.13, and current smoking was also associated with LQTS (aOR 3.71, 95% CI 3.53–3.90), as shown in Table 3.

**TABLE 2:** Associations between number of QT-prolonging drugs and risk of long QT syndrome.

No. of QT-prolonging drugs	Cases (n = 335,693), n (%)	Controls (n = 335,693), n (%)	Crude odds ratio (95% CI)	Adjusted odds ratio (95% CI)
0	282,231 (84.1)	322,810 (96.2)	Reference	Reference
1	35,586 (10.6)	8,709 (2.6)	4.45 (4.35–4.56)	6.70 (6.25–7.17)
2	11,431 (3.4)	2,314 (0.7)	5.12 (4.90–5.36)	11.63 (10.16–13.30)
≥3	6,346 (1.9)	1,860 (0.6)	3.46 (3.28–3.64)	15.08 (11.79–19.30)

Abbreviations: OR, odds ratio; aOR, adjusted odds ratio; CI, confidence interval.

**TABLE 3:** Associations between clinical parameters and risk of long QT syndrome.

Clinical parameters	Cases (n = 335,693), n (%)	Controls (n = 335,693), n (%)	Crude odds ratio (95% CI)	Adjusted odds ratio (95% CI)
BMI ≥ 30 kg/m^2^	20,217 (6.0)	3,343 (1.0)	6.37 (6.14–6.61)	4.93 (4.88–4.97)
WC > 90 cm, male	51,998 (31.9)	8,676 (5.3)	8.34 (8.15–8.55)	6.70 (6.48–6.92)
WC > 85 cm, female	51,466 (29.8)	4,298 (2.5)	16.61 (16.09–17.15)	12.29 (12.09–12.50)
SBP ≥ 140 mmHg	65,607 (19.5)	14,367 (4.3)	5.43 (5.33–5.54)	5.41 (5.26–5.57)
DBP ≥ 90 mmHg	33,906 (10.1)	3,703 (1.1)	10.07 (9.73–10.42)	8.75 (8.37–9.15)
Hgb ≥ 16 g/dL, male	28,723 (17.6)	12,138 (7.4)	2.66 (2.60–2.72)	3.47 (3.36–3.59)
Hgb ≥ 14.5 g/dL, female	16,470 (9.5)	2,428 (1.4)	7.38 (7.07–7.71)	4.68 (4.43–4.94)
FBS ≥ 126 mg/dL	55,710 (16.6)	8,875 (2.6)	7.33 (7.16–7.50)	4.56 (4.51–4.62)
T.chol ≥ 200 mg/dL	96,256 (28.7)	31,448 (9.4)	3.89 (3.84–3.94)	6.85 (6.68–7.02)
TG ≥ 200 mg/dL	41,603 (12.4)	11,899 (3.5)	3.85 (3.77–3.93)	3.23 (3.13–3.33)
HDL-C < 40 mg/dL, male	29,661 (18.2)	6,487 (4.0)	5.37 (5.23–5.53)	4.09 (3.96–4.24)
HDL-C < 50 mg/dL, female	49,276 (28.5)	6,089 (3.5)	10.91 (10.61–11.22)	13.95 (12.39–17.52)
LDL-C ≥ 160 mg/dL	20,085 (6.0)	6,405 (1.9)	3.27 (3.18–3.37)	5.95 (5.74–6.18)
AST ≥ 120 U/L	1,867 (0.6)	677 (0.2)	2.77 (2.53–3.02)	0.68 (0.60–0.76)
ALT ≥ 120 U/L	1,945 (0.6)	398 (0.1)	4.91 (4.41–5.47)	1.39 (1.21–1.60)
γ-GGT ≥ 60 U/L, male	35,968 (22.1)	12,059 (7.4)	3.55 (3.47–3.63)	2.29 (2.22–2.36)
γ-GGT ≥ 40 U/L, female	24,527 (14.2)	2,786 (1.6)	10.08 (9.69–10.49)	6.81 (6.67–6.95)
Scr > 1.3 mg/dL, male	16,366 (10.0)	1,075 (0.7)	16.83 (15.82–17.91)	18.83 (15.49–22.42)
Scr > 1.1 mg/dL, female	10,189 (5.9)	478 (0.3)	22.56 (20.58–24.74)	21.13 (19.02–23.48)
GFR < 60 mL/min/1.73 m2	50,449 (15.0)	3,582 (1.1)	16.40 (15.85–16.97)	16.23 (15.59–16.90)
Smoking^a^	11,697 (18.3)	3,270 (13.3)	3.66 (3.52–3.81)	3.71 (3.53–3.90)

Abbreviations: BMI, body mass index; WC, waist circumference; SBP, systolic blood pressure; DBP, diastolic blood pressure; Hgb, hemoglobin; FBS, fasting blood sugar; T.chol, total cholesterol; TG, triglyceride; HDL-C, high-density lipoprotein cholesterol; LDL-C, low-density lipoprotein cholesterol; AST, aspartate aminotransferase; ALT, alanine aminotransferase; γ-GGT, gamma-glutamyl transferase; Scr, serum creatinine; GFR, glomerular filtration rate; OR, odds ratio; aOR, adjusted odds ratio; CI, confidence interval.

^a^ Smoking indicates current smoking status at baseline.

### Subgroup analyses by sex and age

Subgroup analyses stratified by sex revealed broadly consistent associations between comorbidities and LQTS risk in both men and women, with some differences for metabolic conditions. Hyperkalaemia showed a numerically higher OR in women (21.55, 95% CI 19.54–23.77) than in men (18.80, 17.05–20.74), although confidence intervals overlapped. Both hypothyroidism and thyrotoxicosis were associated with higher risk in women than in men. The number of QT-prolonging drugs was positively associated with risk in both sexes (Supplementary Table S5). Age-stratified analyses showed generally similar patterns of comorbidity-associated risk across age groups. For QT-prolonging drug exposure, the risk associated with taking three or more drugs increased with age, from 5.41 (4.72–6.20) in patients <40 years, to 8.80 (8.15–9.50) in those aged 40–64 years, and 19.01 (16.75–21.40) in those aged ≥65 years (Supplementary Table S6).

## Discussion

This study used a population-based, individually matched case-control design including 671,386 Korean adults in 335,693 matched case–control pairs to evaluate the associations between LQTS and a broad spectrum of potential factors. To our knowledge, this is among the largest population-based analyses of factors associated with LQTS in an Asian adult population within real-world clinical settings, jointly considering comorbidities, cumulative QT-prolonging drug exposure, laboratory parameters, and lifestyle factors. These findings may provide a basis for future region-specific QT prolongation risk models and support pharmacovigilance for QT-prolonging drugs in Korean adults.

Major cardiovascular conditions emerged as strong independent correlates of LQTS, with hypertension, chronic heart failure, and peripheral arterial disease each showing aORs of approximately 20. The magnitude of these associations likely reflects two methodological features. First, cases were identified by clinical LQTS-related diagnostic codes whereas controls were drawn from individuals without any arrhythmia diagnosis, producing a more extreme contrast than studies that compare symptomatic with at-risk cohorts. Second, the baseline prevalence of many comorbidities was very low in the control group, which inflates the magnitude of the resulting odds ratios. These estimates should therefore be interpreted as relative rankings of association strength rather than measures of absolute risk, and direct numerical comparison with effect sizes from other case definitions or sampling frames is not straightforward. With that caveat, the consistent direction and ordering of associations across cardiovascular, endocrine, oncologic, and electrolyte categories support the biological plausibility of these findings, since each of these conditions can promote QT interval prolongation through pathways involving myocardial remodelling, conduction-system disease, autonomic dysregulation, and altered ion-channel function (Gorenek et al., 2024; Crotti et al., 2012).

Among electrolyte abnormalities, hypermagnesaemia, hyperkalaemia, and calcium imbalance were all strongly associated with LQTS, with aORs exceeding 15. Hyperkalaemia, however, was associated with accelerated repolarisation and QT shortening rather than prolongation (Weiss, Qu and Shivkumar, 2017). Thus, this positive association is unlikely to reflect a direct electrophysiological effect of hyperkalemia itself, but may instead reflect confounding by clinical severity. Electrolyte abnormalities in this study were identified using ICD-coded diagnoses rather than serum concentrations measured at the electrocardiographic assessment. These codes likely captured clinically significant disturbances occurring in patients with advanced chronic kidney disease, acute kidney injury, or critical illness, all of which may be associated with QT prolongation through conduction-system remodelling, metabolic instability and coexisting electrolyte abnormalities such as hypocalcaemia (Liu et al., 2020). The comparable association observed for hypermagnesaemia, which is not typically considered a QT-prolonging derangement and for which magnesium is used therapeutically for torsades de pointes (Tzivoni et al., 1988), further supports the interpretation that these codes served mainly as markers of renal dysfunction, clinical severity, and intensified electrocardiographic surveillance rather than as direct causal drivers of repolarization abnormalities (El-Sherif and Turitto, 2011). Ethnic differences in distributions of polymorphisms in repolarizing potassium channel genes such as KCNH2 and in drug-metabolizing enzymes have been reported in East Asian compared with European populations (Seyerle et al., 2014), but a direct contribution of these differences to the magnitudes observed in this study cannot be determined from administrative data alone. Thyroid dysfunction was another notable correlate. Hypothyroidism prolongs the QT interval by downregulating cardiac potassium channels and slowing repolarization, while thyrotoxicosis has also been associated with QTc prolongation through altered ion-channel expression and increased repolarization heterogeneity (Lee et al., 2015). Both extremes of thyroid hormone activity may therefore destabilize cardiac electrophysiology, supporting the relevance of thyroid assessment when stratifying patients for QT prolongation risk.

A particularly notable finding was the graded rise in the strength of association with the number of concurrently administered QT-prolonging drugs. The aOR increased from 6.70 for one drug to 11.63 for two and 15.08 for three or more, with the largest incremental change between zero and two drugs. These estimates were substantially higher than those reported in some Western analyses. A Jordanian intensive-care-unit study reported an OR of 4.28 for the use of more than five medications overall (Al-Azayzih et al., 2024), and an Italian study of geriatric inpatients reported an OR of 2.18 for use of more than two QT-prolonging drugs (Rossi et al., 2021). Because of methodological differences in case definition, control selection, and exposure window, the absolute magnitudes are not directly comparable; the consistent finding across these studies is the direction and steepness of the dose-response relationship rather than the exact OR value. The steep gradient in our data may reflect a combination of additive ion-channel blockade, pharmacokinetic interactions raising plasma concentrations, and drug-induced electrolyte disturbance (Roden, 2008). Pharmacogenetic variation may also contribute to East–West differences, as the distribution of polymorphisms in drug-metabolizing enzymes (*CYP2D6, CYP3A5, CYP2C19*) and ion-channel genes (*KCNH2, SCN5A, KCNQ1*) differs between East Asian and European populations (Iwasa et al., 2000). Antiarrhythmic agents showed the highest aOR (9.29), followed by antidepressants (8.47) and urologic agents (7.80); these orderings broadly align with classes implicated in earlier population-based analyses (Eroglu et al., 2022), although the absolute magnitudes in our cohort were larger for the methodological reasons outlined above.

Several health screening parameters were also associated with LQTS, including central obesity, elevated blood pressure on screening, dyslipidaemia, and renal dysfunction. Renal dysfunction in particular showed one of the highest associations, consistent with the established roles of impaired drug clearance and disordered electrolyte handling in cardiac repolarization. Current smoking was independently associated with LQTS (aOR 3.71), in line with experimental evidence that nicotine and other tobacco-related exposures increase dispersion of ventricular repolarization (D'Alessandro et al., 2012).

In sex-stratified analysis, broad patterns of association were similar between men and women, with some numerical differences in metabolic and endocrine variables. Hyperkalaemia showed a higher point estimate in women (OR 21.55) than in men (OR 18.80), although the 95% confidence intervals overlapped substantially; this should therefore be regarded as a hypothesis-generating observation rather than a confirmed sex difference. Both hypothyroidism and thyrotoxicosis showed higher point estimates in women than in men. Sex differences in cardiac repolarization, mediated in part by oestrogen and testosterone effects on ion-channel expression, are well documented and may contribute to these patterns (Stramba-Badiale et al., 1997). Diuretic-induced electrolyte disturbance has also been reported more frequently in women in Japanese claims data (Maida et al., 2025), which may add to susceptibility in older female patients.

In age-stratified analysis, the aOR for taking three or more QT-prolonging drugs increased with age, reaching 19.01 in adults aged ≥65 years. This pattern is consistent with well-recognized age-related reductions in hepatic and renal drug clearance and the accumulation of comorbidities. Direct numerical comparisons with previously reported elderly cohorts are limited by differences in case definition, but the steep age gradient in our data underscores the relevance of structured medication review and ECG monitoring in older Korean adults on multiple QT-prolonging drugs. Together, these findings may complement existing decision-support tools such as CredibleMeds (Then et al., 2022), which were largely derived from Western populations and clinical settings, by providing population-specific information for Korean adults.

A few analytical choices warrant comment. Because our aim was to identify and rank a broad panel of comorbidity, drug, and laboratory factors rather than to estimate a single exposure effect, we matched only on age and sex and adjusted for all remaining covariates through conditional logistic regression; matching those factors through a propensity score would induce overmatching and remove them from the estimable set, although a targeted propensity score would be well suited to future analyses centred on a single QT-prolonging drug. Consistent with this objective, we relied on direct, simultaneous adjustment for all measured covariates through multivariable conditional logistic regression, which preserves an interpretable, factor-level adjusted odds ratio for each comorbidity, drug, and laboratory abnormality—the quantities of primary interest for the pharmacovigilance aim of this study—and, given the large sample size, is not constrained by model dimensionality. The descriptive co-occurrence patterns shown in Figure 2 may also inform future studies aimed at identifying distinct multimorbidity phenotypes using unsupervised methods, such as clustering.

Several limitations should be acknowledged. First, cases were defined using ICD-10 diagnostic codes for LQTS and related arrhythmias together with a claim for an electrocardiographic procedure; QTc measurements themselves were not available. Use of administrative codes likely identifies a subset of clinically symptomatic or actively investigated cases and may miss asymptomatic prolongation. Moreover, the diagnostic code set spans congenital long QT syndrome, acquired QT prolongation, and downstream arrhythmias (torsades de pointes, ventricular fibrillation), so the phenotype is heterogeneous, chronicity and the congenital-versus-acquired distinction cannot be verified, and the positive predictive value of a claims-based algorithm for conduction abnormalities remains uncertain (Ye et al., 2018). Future studies integrating hospital-based electronic medical records that include numerical QTc values would allow more precise phenotyping. Second, controls were defined as individuals without any arrhythmia diagnosis. This produces a contrast between a clinically diagnosed group and a relatively healthy reference group, which inflates the magnitude of odds ratios for many comorbidities and limits the interpretation of effect sizes as absolute measures of risk. Sensitivity analyses using alternative control selections (for example, restricting cases to a single ICD code or using healthcare-encounter-matched controls) would help characterize the size of this bias. Third, confounding by indication cannot be fully excluded: patients with multiple comorbidities are also more likely to be prescribed multiple QT-prolonging drugs, so part of the observed drug-class associations may reflect the underlying conditions for which the drugs were prescribed. Fourth, drug exposure was based on filled prescriptions during the 1 month preceding the index date, treated as a binary variable; this does not capture dose, duration, or concurrent overlap precisely. Relatedly, electrolyte disturbances were captured as diagnostic codes rather than serum concentrations measured at the time of electrocardiography, so the direction of any electrophysiological effect cannot be established and these associations should be read as markers of clinical severity and detection context rather than direct causal effects. Fifth, several covariates are correlated—for example, ICD-10–coded hypertension and screening blood pressure, or serum creatinine and estimated glomerular filtration rate—and simultaneous adjustment for coded hypertension and measured blood pressure in particular may lead to over-adjustment; given the large sample size, however, such collinearity chiefly widens confidence intervals rather than biasing point estimates, and results for these correlated variables should be interpreted with this in mind (Vatcheva et al., 2016). Sixth, smoking status was self-reported on the screening questionnaire, and lifestyle exposures such as alcohol use, physical activity, and diet were not included in structured form, raising the possibility of residual confounding. Finally, although the cohort represents Korean adults under universal health insurance, the findings may not generalize to other East Asian populations or to clinical settings outside Korea.

In a large Korean adult population, clinically diagnosed LQTS was strongly associated with cardiovascular, endocrine, and electrolyte-related comorbidities, and the strength of association rose steeply with the number of concurrently administered QT-prolonging drugs. Older adults and patients with electrolyte abnormalities such as hyperkalaemia or calcium imbalance showed particularly strong associations and may merit closer monitoring for QT prolongation when QT-prolonging medication is prescribed. While effect sizes should be interpreted as relative rankings rather than absolute risk estimates, the consistency and direction of these associations support their use, alongside existing tools such as CredibleMeds, in informing population-specific approaches to QT prolongation risk assessment in Korean adults.

## Data Availability

The data analyzed in this study is subject to the following licenses/restrictions: The data are not publicly available. They were obtained from the Korean National Health Insurance Service (NHIS) under a data use agreement and contain sensitive health information. Researchers can request access through the National Health Insurance Sharing Service portal (https://nhiss.nhis.or.kr), subject to NHIS approval and applicable data protection regulations. Analytic code is available from the corresponding authors on reasonable request. Requests to access these datasets should be directed to https://nhiss.nhis.or.kr.
