# Supplementary material for: Factors associated with clinically diagnosed long QT syndrome in Korean adults: a nationwide case-control study
Source: Front Pharmacol. 2026 Aug 20;17:1904560. doi: 10.3389/fphar.2026.1904560 (PMC13538420; doi:10.3389/fphar.2026.1904560)
Supplement: Supplementary file 1 [file Supplementaryfile1.docx]

**Supplementary Material**

Supplementary Table S1. ICD-10 codes of comorbidities

Supplementary Table S2. QT-prolonging medications and severity of QT risk

Supplementary Table S3. Baseline characteristics of the study population

Supplementary Table S4. Association between QT-drug classes and the risk of long QT syndrome

Supplementary Table S5. Risk factors for long QT syndrome according to sex

Supplementary Table S6. Risk factors for long QT syndrome according to age groups

Supplementary Figure S1. Study timeline for covariate assessment and drug exposure windows

Supplementary references

# Supplementary Table S1. ICD-10 codes of comorbidities

| Comorbidities | ICD-10 codes |
| --- | --- |
| Hypertension | I10.*, I15.* |
| Myocardial infarction | I21.*, I22.*, I25.2 |
| Chronic heart failure | I09.9, I11.0, I13.0, I13.2, I25.5, I42.0, I42.5−I42.9, I43.*, I50.*, P29.0 |
| Diabetes mellitus | E10.*−E14.* |
| Chronic kidney diseases | I12.0, N03.1−N03.7, N05.2−N05.7, N18.*, N19.*, N25.0, Z49.0−Z49.2, Z94.0, Z99.2 |
| Hypothyroidism | E00.*−E03.*, E89 |
| Thyrotoxicosis | E05, E06.2 |
| Cancers | C00.*−C97.* |
| Cerebrovascular diseases | G45.*, G46.*, I60.*−I69.*, H34.0 |
| Peripheral arterial diseases | I70.*, I71.*, I73.1, I73.8, I73.9, I77.1, I79.0, I79.2, K55.1, K55.8, K55.9, Z95.8, Z95.9 |
| Atrial fibrillation | I48.0−I48.4, I48.9 |
| Hypermagnesemia | E83.4 |
| Hypomagnesemia | E61.2 |
| Hyperkalaemia | E87.5 |
| Hypokalaemia | E87.6 |
| Calcium imbalance | E83.5 |

Abbreviations: ICD-10, International Classification of Diseases, 10th Revision.

# Supplementary Table S2. QT-prolonging drugs and their severity of QT Risk

| Drug classes | Medications | Severity of QT risk | References |
| --- | --- | --- | --- |
| Analgesics | Oxycodone | Not classified | 1 |
|  | Buprenorphine | Possible risk of TdP | 2, 3 |
| Antiarrhythmics | Amiodarone | Known risk of TdP | 3, 4 |
|  | Flecainide | Known risk of TdP | 3, 5 |
|  | Propafenone | Conditional risk of TdP | 3, 6 |
|  | Dobutamine | Special risk for patients with congenital long QT | 3 |
|  | Dronedarone | Known risk of TdP | 3, 7 |
|  | Sotalol | Known risk of TdP | 3, 8 |
| Antidepressants | Escitalopram | Known risk of TdP | 3, 7 |
|  | Amitriptyline | Conditional risk of TdP | 3, 7 |
|  | Trazodone | Conditional risk of TdP | 3, 9 |
|  | Paroxetine | Conditional risk of TdP | 3, 10 |
|  | Mirtazapine | Possible risk of TdP | 3, 11 |
|  | Sertraline | Conditional risk of TdP | 3, 12 |
|  | Fluoxetine | Conditional risk of TdP | 3, 12 |
|  | Imipramine | Possible risk of TdP | 3, 7 |
|  | Venlafaxine | Possible risk of TdP | 3, 13 |
|  | Doxepin | Conditional risk of TdP | 3, 14 |
|  | Desvenlafaxine | Not classified | 15 |
| Antiemetics | Metoclopramide | Conditional risk of TdP | 3, 12 |
|  | Domperidone | Known risk of TdP | 3, 7 |
|  | Ondansetron | Known risk of TdP | 3, 7 |
| Antifungals | Fluconazole | Known risk of TdP | 3, 16 |
|  | Voriconazole | Possible risk of TdP | 3, 16 |
| Antiplatelets | Cilostazol | Known risk of TdP | 3, 8 |
| Antipsychotics | Risperidone | Conditional risk of TdP | 3, 17 |
|  | Haloperidol | Known risk of TdP | 3, 7 |
|  | Aripiprazole | Possible risk of TdP | 3, 18 |
|  | Perphenazine | Possible risk of TdP | 3, 17 |
|  | Olanzapine | Conditional risk of TdP | 3, 17 |
|  | Chlorpromazine | Known risk of TdP | 3, 7 |
|  | Clozapine | Possible risk of TdP | 3, 17 |
|  | Amisulpride | Conditional risk of TdP | 3, 7 |
|  | Paliperidone | Possible risk of TdP | 3 |
|  | Ziprasidone | Conditional risk of TdP | 3, 17 |
|  | Pimozide | Known risk of TdP | 3, 7 |
| Bronchodilators | Salbutamol | Special risk for patients with congenital long QT | 3, 19 |
|  | Formoterol | Special Risk for patients with congenital long QT | 3, 20 |
| Cardiac glycosides | Digoxin | Not classified | 21 |
| Cognitive enhancers | Donepezil | Known risk of TdP | 3,22 |
|  | Galantamine | Conditional risk of TdP | 3, 23 |
|  | Amantadine | Conditional risk of TdP | 3 |
| Diuretics | Furosemide | Conditional risk of TdP | 3, 18 |
|  | Hydrochlorothiazide | Conditional risk of TdP | 3, 18 |
| Macrolides | Clarithromycin | Known risk of TdP | 3, 7 |
|  | Azithromycin | Known risk of TdP | 3, 7 |
| Other antibiotics | Amikacin | Not classified | 24 |
|  | Metronidazole | Conditional risk of TdP | 3 |
| Quinolones | Levofloxacin | Known risk of TdP | 3, 7 |
|  | Ciprofloxacin | Known risk of TdP | 3, 8 |
|  | Ofloxacin | Possible risk of TdP | 3 |
|  | Moxifloxacin | Known risk of TdP | 3, 7 |
|  | Gemifloxacin | Possible risk of TdP | 3 |
| Urologicals | Alfuzosin | Possible risk of TdP | 3 |
|  | Solifenacin | Conditional risk of TdP | 3 |
|  | Tolterodine | Possible risk of TdP | 3, 12 |

Abbreviations: TdP, torsades de pointes.

Supplementary Table S3. Baseline characteristics of the study population

| Characteristics | Cases (n = 335,693) | Controls (n = 335,693) | SMD |
| --- | --- | --- | --- |
| Sex, male, n (%) | 162,983 (48.6) | 163,197 (48.6) | 0.0013 |
| Age, mean ± SD | 54.6 ± 19.1 | 54.6 ± 19.1 | 0.0008 |
| <40 years, n (%) | 96,904 (28.9) | 97,053 (28.9) | 0.0010 |
| 40–64 years, n (%) | 87,110 (25.9) | 87,066 (25.9) | 0.0028 |
| ≥65 years, n (%) | 151,679 (45.2) | 151,574 (45.2) | 0.0030 |

Abbreviations: SMD, standardized mean difference.

Supplementary Table S4. Association between QT-drug classes and risk of long QT syndrome

| Drug class | Cases (n = 335,693), n (%) | Controls (n = 335,693), n (%) | Crude odds ratio (95% CI) | Adjusted odds ratio (95% CI) |
| --- | --- | --- | --- | --- |
| Analgesics | 1,105 (0.3) | 276 (0.1) | 4.01 (3.52–4.58) | 4.35 (3.70–5.13) |
| Antiarrhythmics | 7,353 (2.2) | 858 (0.3) | 8.74 (8.14–9.38) | 9.29 (8.56–10.10) |
| Antidepressants | 12,445 (3.7) | 1,654 (0.5) | 7.78 (7.38–8.19) | 8.47 (7.96–9.01) |
| Antiemetics | 8,626 (2.6) | 3,278 (1.0) | 2.67 (2.57–2.79) | 3.68 (3.47–3.90) |
| Antifungals | 2,009 (0.6) | 919 (0.3) | 2.19 (2.03–2.37) | 3.14 (2.81–3.52) |
| Antiplatelets | 2,221 (0.7) | 308 (0.1) | 7.25 (6.44–8.17) | 3.73 (3.22–4.33) |
| Antipsychotics | 3,815 (1.1) | 1,581 (0.5) | 2.43 (2.29–2.58) | 2.82 (2.61–3.05) |
| Bronchodilators | 2,202 (0.7) | 838 (0.3) | 2.64 (2.44–2.86) | 3.03 (2.73–3.36) |
| Cardiac glycosides | 3,612 (1.1) | 323 (<0.1) | 11.29 (10.08–12.66) | 2.18 (1.47–2.19) |
| Cognitive enhancers | 3,508 (1.1) | 962 (0.3) | 3.67 (3.42–3.95) | 5.36 (4.86–5.94) |
| Diuretics | 13,652 (4.1) | 3,635 (1.1) | 3.87 (3.73–4.02) | 4.01 (3.47–4.34) |
| Macrolides | 3,027 (0.9) | 1,555 (0.5) | 1.96 (1.84–2.08) | 2.63 (2.40–2.89) |
| Other antibiotics | 545 (0.2) | 437 (0.1) | 1.25 (1.10–1.41) | 1.42 (1.19–1.70) |
| Quinolones | 6,721 (2.0) | 3,561 (1.1) | 1.91 (1.83–1.99) | 2.69 (2.53–2.86) |
| Urologicals | 2,230 (0.7) | 323 (0.1) | 6.94 (6.18–7.80) | 7.80 (6.80–8.94) |

**Abbreviations:** OR, odds ratio; aOR, adjusted odds ratio; CI, confidence interval.

Supplementary Table S5. Risk factors for long QT syndrome according to sex

| Variable | Male (n = 326,180) | | | Female (n =345,206) | | |
| --- | --- | --- | --- | --- | --- | --- |
|  | Case (n = 162,983), n (%) | Control (n = 163,197), n (%) | aOR (95% CI) | Case (n = 172,710), n (%) | Control (n = 172,496), n (%) | aOR (95% CI) |
| Comorbidities | | | | | | |
| Hypertension | 77,892 (47.8) | 6,084 (3.7) | 19.74 (15.23–23.86) | 76,879 (44.5) | 5,425 (3.1) | 19.22 (16.49–22.23) |
| Myocardial infarction | 7,184 (4.4) | 610 (0.4) | 11.71 (10.52–14.01) | 4,279 (2.5) | 244 (0.1) | 12.71 (11.05–19.53) |
| Chronic heart failure | 27,261 (16.7) | 1,329 (0.8) | 19.74 (16.97–22.81) | 25,011 (14.5) | 886 (0.5) | 22.01 (19.93–24.41) |
| Cerebrovascular diseases | 20,185 (12.4) | 1,986 (1.2) | 9.01 (7.79–10.34) | 20,768 (12.0) | 1,578 (0.9) | 10.37 (8.94–11.96) |
| Peripheral arterial diseases | 20,751 (12.7) | 864 (0.5) | 21.34 (18.42–24.57) | 23,253 (13.5) | 954 (0.6) | 23.88 (21.18–25.85) |
| Diabetes mellitus | 45,392 (27.9) | 4,422 (2.7) | 13.24 (11.26–14.42) | 41,729 (24.2) | 2,999 (1.7) | 12.52 (11.79–13.43) |
| Hypothyroidism | 7,079 (4.3) | 370 (0.2) | 16.59 (14.67–19.71) | 13,997 (8.1) | 623 (0.4) | 18.49 (16.21–21.01) |
| Thyrotoxicosis | 3,905 (2.4) | 282 (0.2) | 10.34 (8.01–12.81) | 6,451 (3.7) | 354 (0.2) | 13.70 (11.95–15.62) |
| Cancers | 15,584 (9.6) | 1,795 (1.1) | 7.41 (6.44–8.48) | 12,217 (7.1) | 1,211 (0.7) | 7.77 (6.77–8.88) |
| Chronic kidney diseases | 6,384 (3.9) | 433 (0.3) | 11.74 (9.27–14.36) | 4,056 (2.3) | 364 (0.2) | 10.32 (8.26–12.48) |
| Hypermagnesemia | 158 (0.1) | 12 (0.0) | 12.74 (10.74–14.85) | 193 (0.1) | 5 (0.0) | 18.29 (16.12–20.69) |
| Hypomagnesemia | 187 (0.1) | 18 (0.0) | 9.10 (8.25–10.04) | 309 (0.2) | 24 (0.0) | 11.56 (10.48–12.75) |
| Hyperkalaemia | 2,947 (1.8) | 159 (0.1) | 18.80 (17.05–20.74) | 2,068 (1.2) | 94 (0.1) | 21.55 (19.54–23.77) |
| Hypokalaemia | 1,555 (1.0) | 174 (0.1) | 8.97 (8.13–9.89) | 1,915 (1.1) | 183 (0.1) | 10.45 (9.47–11.52) |
| Calcium imbalance | 2,201 (1.4) | 144 (0.1) | 15.35 (13.92–16.93) | 4,073 (2.4) | 129 (0.1) | 20.45 (18.52–22.69) |
| Number of QT drugs | | | | | | |
| 1 | 16,743 (10.3) | 4,484 (2.7) | 6.52 (5.89–7.22) | 18,843 (10.9) | 4,225 (2.4) | 6.50 (5.87–7.20) |
| 2 | 5,446 (3.3) | 1,270 (0.8) | 9.37 (7.88–11.19) | 5,985 (3.5) | 1,044 (0.6) | 10.74 (8.78–13.14) |
| ≥3 | 3,037 (1.9) | 1,064 (0.7) | 13.40 (9.30–19.30) | 3,309 (1.9) | 796 (0.5) | 15.53 (11.57–20.83) |

Abbreviations: aOR, adjusted odds ratio; CI, confidence interval.

Supplementary Table S6. Risk factors for long QT syndrome according to age groups

| Variable | Age <40 years (n = 193,957) | | | Age 40–65 years (n = 174,176) | | | Age ≥65 years (n = 303,253) | | |
| --- | --- | --- | --- | --- | --- | --- | --- | --- | --- |
|  | Case (n = 96,904), n (%) | Control (n = 97,053), n (%) | aOR (95% CI) | Case (n = 87,110), n (%) | Control (n = 87,066), n (%) | aOR (95% CI) | Case (n = 151,679), n (%) | Control (n = 151,574), n (%) | aOR (95% CI) |
| Comorbidities | | | | | | | | | |
| Hypertension | 2,269 (2.3) | 167 (0.2) | 18.19 (16.12–19.62) | 34,809 (40.0) | 2,805 (3.2) | 21.21 (19.79–22.70) | 117,693 (77.6) | 8,537 (5.6) | 22.71 (20.53–24.44) |
| Myocardial infarction | 305 (0.3) | 16 (0.0) | 15.18 (11.60–19.72) | 2,685 (3.1) | 239 (0.3) | 11.86 (10.38–13.55) | 8,473 (5.6) | 599 (0.4) | 15.41 (14.18–16.75) |
| Chronic heart failure | 1,620 (1.7) | 81 (0.1) | 20.58 (16.46–25.72) | 11,408 (13.1) | 561 (0.6) | 21.89 (19.67–24.30) | 39,244 (25.9) | 1,573 (1.0) | 23.12 (21.31–25.02) |
| Cerebrovascular diseases | 500 (0.5) | 43 (0.0) | 11.74 (8.59–16.03) | 6,082 (7.0) | 636 (0.7) | 10.88 (10.02–11.82) | 34,371 (22.7) | 2,885 (1.9) | 12.35 (11.77–13.96) |
| Peripheral arterial diseases | 624 (0.6) | 36 (0.0) | 17.55 (12.54–24.56) | 8,261 (9.5) | 314 (0.4) | 21.97 (18.54–25.81) | 35,119 (23.2) | 1,468 (1.0) | 22.30 (21.69–23.60) |
| Diabetes mellitus | 1,510 (1.6) | 162 (0.2) | 9.58 (8.15–11.27) | 19,102 (21.9) | 2,054 (2.4) | 11.44 (10.71–12.20) | 66,509 (43.9) | 5,205 (3.4) | 13.16 (12.35–14.00) |
| Hypothyroidism | 1,064 (1.1) | 92 (0.1) | 11.80 (9.53–14.60) | 6,483 (7.4) | 385 (0.4) | 19.48 (17.56–21.61) | 13,529 (8.9) | 516 (0.3) | 21.68 (19.26–23.33) |
| Thyrotoxicosis | 981 (1.0) | 87 (0.1) | 11.48 (9.22–14.30) | 3,954 (4.5) | 282 (0.3) | 13.24 (11.50–15.21) | 5,421 (3.6) | 267 (0.2) | 16.16 (13.71–18.93) |
| Cancers | 509 (0.5) | 50 (0.1) | 7.28 (4.68–10.75) | 6,513 (7.5) | 828 (0.9) | 8.02 (7.38–8.71) | 20,779 (13.7) | 2,128 (1.4) | 9.71 (9.19–11.25) |
| Chronic kidney diseases | 118 (0.1) | 22 (0.0) | 5.39 (3.42–8.50) | 1,927 (2.2) | 186 (0.2) | 10.76 (9.25–12.52) | 8,395 (5.5) | 589 (0.4) | 12.28 (11.05–13.61) |
| Hypermagnesemia | 24 (0.0) | 1 (0.0) | 14.96 (3.26–191.21) | 100 (0.1) | 8 (0.0) | 12.50 (6.08–25.68) | 227 (0.2) | 8 (0.0) | 18.44 (14.05–57.58) |
| Hypomagnesemia | 23 (0.0) | 2 (0.0) | 11.53 (2.72–48.97) | 139 (0.2) | 7 (0.0) | 12.09 (9.37–43.07) | 334 (0.2) | 33 (0.0) | 11.14 (7.09–14.49) |
| Hyperkalaemia | 54 (0.1) | 8 (0.0) | 6.76 (3.22–14.20) | 918 (1.1) | 58 (0.1) | 16.11 (12.35–21.01) | 4,043 (2.7) | 187 (0.1) | 17.33 (14.28–21.86) |
| Hypokalaemia | 89 (0.1) | 4 (0.0) | 12.55 (8.24–61.75) | 596 (0.7) | 94 (0.1) | 6.40 (5.15–7.96) | 2,785 (1.8) | 259 (0.2) | 8.93 (7.63–12.42) |
| Calcium imbalance | 200 (0.2) | 19 (0.0) | 15.54 (11.59–18.87) | 1,626 (1.9) | 93 (0.1) | 18.07 (14.66–22.28) | 4,448 (2.9) | 161 (0.1) | 18.40 (14.27–23.24) |
| Number of QT drugs | | | | | | | | | |
| 1 | 2,229 (2.3) | 978 (1.0) | 1.76 (1.68–1.84) | 8,965 (10.3) | 3,159 (3.6) | 2.06 (2.01–2.12) | 24,392 (16.1) | 4,572 (3.0) | 3.39 (3.32–3.46) |
| 2 | 605 (0.6) | 193 (0.2) | 3.08 (2.81–3.38) | 2,536 (2.9) | 746 (0.9) | 4.26 (4.05–4.49) | 8,290 (5.5) | 1,375 (0.9) | 11.50 (11.05–11.97) |
| ≥3 | 323 (0.3) | 151 (0.2) | 5.41 (4.72–6.20) | 1,219 (1.4) | 631 (0.7) | 8.80 (8.15–9.50) | 4,804 (3.2) | 1,078 (0.7) | 19.01 (16.75–21.40) |

**Abbreviations:** aOR, adjusted odds ratio; CI, confidence interval.

**Supplementary Figure S1. Study timeline for covariate assessment and drug exposure windows.** The comorbidity assessment window covered the 6 months prior to the index date. Drug exposure was assessed during the 1 month prior to the index date and includes prescriptions with end dates falling within this period. The nearest prior health checkup date to the index date is shown. The dashed vertical line indicates the index date (LQTS diagnosis).

# **Supplementary references**

1. Fanoe S, Jensen GB, Sjogren P, et al. Oxycodone is associated with dose-dependent QTc prolongation in patients and low-affinity inhibiting of hERG activity in vitro. Br J Clin Pharmacol. 2009;67(2):172–9.

2. Isbister GK, Brown AL, Gill A, et al. QT interval prolongation in opioid agonist treatment: analysis of continuous 12-lead electrocardiogram recordings. Br J Clin Pharmacol. 2017;83(10):2274–82.

3. Then MI, Andrikyan W, Maas R, et al. The CredibleMeds^®^ list: Usage of QT interval prolonging drugs in Germany and discordances with prescribing information. Br J Clin Pharmacol. 2022;88(1):226–36.

4. Khatib R, Sabir FRN, Omari C, et al. Managing drug-induced QT prolongation in clinical practice. Postgrad Med J. 2021;97(1149):452–8.

5. Acosta-Materan C, Diaz-Oliva E, Fernandez-Rodriguez D, et al. QT interval prolongation and torsade de pointes: Synergistic effect of flecainide and H1 receptor antagonists. J Pharmacol Pharmacother. 2016;7(2):102–5.

6. Puljevic D, Smalcelj A, Durakovic Z, et al. The influence of atenolol and propafenone on QT interval dispersion in patients 3 months after myocardial infarction. Int J Clin Pharmacol Ther. 1997;35(9):381–4.

7. Thomas SH, Behr ER. Pharmacological treatment of acquired QT prolongation and torsades de pointes. Br J Clin Pharmacol. 2016;81(3):420–7.

8. Li M, Ramos LG. Drug-Induced QT Prolongation And Torsades de Pointes. P T. 2017;42(7):473–7.

9. Khederlou H, Azimi Pirsaraei V. Torsades de Pointe Associated with Trazodone Consumption. Case Rep Crit Care. 2024;2024:5759229. doi:10.1155/2024/5759229

10. Otsuka Y. Paroxetine-induced QTc prolongation. J Gen Fam Med. 2017;18(6):442–5.

11. Allen ND, Leung JG, Palmer BA. Mirtazapine's effect on the QT interval in medically hospitalized patients. Ment Health Clin. 2020;10(1):30–3.

12. Snitker S, Doerfler RM, Soliman EZ, et al. Association of QT-Prolonging Medication Use in CKD with Electrocardiographic Manifestations. Clin J Am Soc Nephrol. 2017;12(9):1409–17.

13. Letsas K, Korantzopoulos P, Pappas L, et al. QT interval prolongation associated with venlafaxine administration. Int J Cardiol. 2006;109(1):116–7.

14. Baker B, Dorian P, Sandor P, et al. Electrocardiographic effects of fluoxetine and doxepin in patients with major depressive disorder. J Clin Psychopharmacol. 1997;17(1):15–21.

15. Aronow WS, Shamliyan TA. Effects of antidepressants on QT interval in people with mental disorders. Arch Med Sci. 2020;16(4):727–41.

16. Kitaya S, Nakano M, Katori Y, Yasuda S, Kanamori H. QTc Interval Prolongation as an Adverse Event of Azole Antifungal Drugs: Case Report and Literature Review. Microorganisms. 2024;12(8):1619. doi:10.3390/microorganisms12081619

17. Stollberger C, Huber JO, Finsterer J. Antipsychotic drugs and QT prolongation. Int Clin Psychopharmacol. 2005;20(5):243–51.

18. Buss VH, Lee K, Naunton M, et al. Identification of Patients At-Risk of QT Interval Prolongation during Medication Reviews: A Missed Opportunity? J Clin Med. 2018;7(12):533. doi:10.3390/jcm7120533

19. Elgassim M, Abdelrahman A, Saied ASS, et al. Salbutamol-Induced QT Interval Prolongation in a Two-Year-Old Patient. Cureus. 2022;14(2):e21904. doi:10.7759/cureus.21904

20. Ferguson GT, Reisner C, Pearle J, et al. Cardiovascular safety profile of a fixed-dose combination of glycopyrrolate and formoterol fumarate delivered via metered dose inhaler using co-suspension delivery technology. Pulm Pharmacol Ther. 2018;49:67–74.

21. Ritter JM. Cardiac safety, drug-induced QT prolongation and torsade de pointes (TdP). Br J Clin Pharmacol. 2012;73(3):331–4.

22. Vogel SM, Mican LM, Smith TL. Donepezil-induced QTc prolongation: A case report. Ment Health Clin. 2019;9(3):128–32.

23. Fisher AA, Davis MW. Prolonged QT interval, syncope, and delirium with galantamine. Ann Pharmacother. 2008;42(2):278–83.

24. Teng C, Walter EA, Gaspar DKS, et al. Torsades de pointes and QT prolongation Associations with Antibiotics: A Pharmacovigilance Study of the FDA Adverse Event Reporting System. Int J Med Sci. 2019;16(7):1018–22.
